# Targeting muscarinic receptors for treating schizophrenia

**DOI:** 10.1016/j.neurot.2026.e00839

**Published:** 2026-02-02

**Authors:** Steven M. Paul, Samantha E. Yohn

**Affiliations:** a Departments of Psychiatry and Neurology, Washington University of St. Louis School of Medicine, USA; b Independent Scholar, Boston, MA, USA

**Keywords:** Schizophrenia, Muscarinic acetylcholine receptors, M_1_ receptors, M_4_ receptors, Xanomeline and trospium, Novel antipsychotic mechanisms

## Abstract

Schizophrenia is a chronic, disabling and potentially fatal psychiatric syndrome characterized by three primary symptom domains: positive, negative, and cognitive symptoms, for which current dopamine D_2_ receptor antagonists provide only partial benefit and are limited by significant side effects. Muscarinic acetylcholine receptors (mAChRs), broadly expressed across cortical, striatal, and midbrain circuits, have emerged as promising targets for next-generation therapies. Among these, M_1_ and M_4_ receptor subtypes play key roles in regulating glutamatergic and dopaminergic transmission. Clinical studies with xanomeline, an orthosteric agonist with functional preference for M_1_ and M_4_ receptors, provided the first proof that mAChR agonists can reduce psychotic symptoms. Reformulation of xanomeline with trospium chloride, a peripherally-restricted mAChR antagonist, improved its tolerability and allowed confirmation of its efficacy in large Phase 2 and 3 trials. Current and future efforts are now focused on developing more selective orthosteric and allosteric mAChR agonists and more precisely characterizing their therapeutic activity (efficacy and safety) in clinical trials. These advances highlight mAChR pharmacology as a novel and clinically validated strategy that extends beyond dopamine D_2_ receptor antagonism to potentially address the full spectrum of schizophrenia symptoms.

## Introduction

More effective and better-tolerated treatments for major psychiatric disorders such as schizophrenia are desperately needed. Current treatments for schizophrenia are only partially effective for most patients, and approximately one-third are considered treatment-resistant [1,2]. Traditional antipsychotic drugs (APDs) are associated with troublesome side effects that contribute to poor adherence [3] and subsequent relapse; moreover, they can increase morbidity and mortality due to iatrogenic illnesses such as morbid obesity, dyslipidemia, diabetes, and hypertension, which heighten the risk of death from stroke or myocardial infarction [[4], [5], [6]]. Although traditional APDs are modestly effective at treating the psychotic (positive) symptoms of schizophrenia, they are relatively ineffective at addressing the negative [7] and cognitive symptoms [8], which are arguably the most disabling aspects of the disorder [[9], [10], [11]]. Despite this substantial unmet medical need, the introduction of more effective and better tolerated APDs has been slow and disappointing. Until very recently, all approved APDs (dating back to the introduction of chlorpromazine in the United States (US) in the early 1950s) have worked primarily by blocking dopamine (DA) D_2_ receptors to control positive symptoms [12,13]. While more than 30 APDs have been marketed to date, with few exceptions, they provide similar efficacy and differ mainly in their side-effect profiles [[14], [15], [16]]. Thus, mechanistically novel APDs, especially those that can beneficially impact negative and cognitive symptoms (in addition to positive symptoms) while avoiding the undesirable side effects of traditional APDs, such as sedation, weight gain, extrapyramidal motor symptoms (EPS)/akathisia, and the risk of developing tardive dyskinesia, are urgently needed.

In September of 2024 the US Food and Drug Administration approved the first in a new class of antipsychotic medicines [17], xanomeline and trospium chloride (trospium; COBENFY™ [brand name]), which was shown in three double blind placebo-controlled trials (Randomized Control Trials [RCTs]) to reduce both the positive and negative symptoms of schizophrenia as measured by the Positive and Negative Symptom Scale (PANSS) total score [[18], [19], [20]]. The efficacy and safety of xanomeline-trospium was also maintained in much longer open label extensions of the acute 5-week placebo-controlled trials ([21] see below for further details). Across all trials, side effects of xanomeline-trospium were primarily cholinergic in nature (both pro- and anticholinergic) and the drug was reasonably well tolerated [[18], [19], [20], [21]]. In this review, we outline the history behind the discovery and development of this novel medicine for schizophrenia, discuss its proposed molecular, cellular, and circuit-level mechanisms of action, and highlight the broader implications for treating not only psychosis but the full spectrum of schizophrenia symptoms, as well as for guiding the design of next-generation muscarinic acetylcholine receptor (mAChR) agents.

## Discovery and Development of Xanomeline and Xanomeline-Trospium (COBENFY™)

In the early 1990s, several pharmaceutical companies began developing drugs that targeted receptors for acetylcholine (ACh) as a way to improve cognition in patients with Alzheimer's disease (AD) and other dementias [22]. In these conditions, cholinergic signaling is disrupted either because the neurons that release ACh degenerate or because the receptors that respond to it function abnormally [23]. This loss of cholinergic signaling contributes to impaired attention, memory formation, and cognitive reserve [24].

Two main strategies were pursued. The first focused on inhibiting acetylcholinesterase [25,26], the enzyme that breaks down ACh, thereby boosting synaptic levels of the neurotransmitter. By increasing the amount of ACh available, both families of ACh receptors, nicotinic receptors (nAChRs; ligand-gated ion channels) and mAChR (G-protein–coupled receptors), could be more strongly stimulated to enhance cognition. This approach led to the development of several cholinesterase inhibitors [27,28], such as donepezil and galantamine, which are still used today to provide modest cognitive benefits in patients with AD [29].

Recognizing that cholinergic neurons themselves eventually degenerate in AD [30], a second approach centered on directly stimulating cholinergic receptors rather than merely increasing synaptic ACh. In particular, mAChR agonists were identified as agents that could activate postsynaptic receptors even in the setting of reduced presynaptic ACh release [31]. Over several decades, abundant preclinical and clinical investigations established a critical role for mAChRs in learning and memory across multiple species, including humans, laying the foundation for their continued exploration as therapeutic targets for dementia (see Refs. [32,33] for review).

### Early work on xanomeline at Eli Lilly

**Fig. 1:**
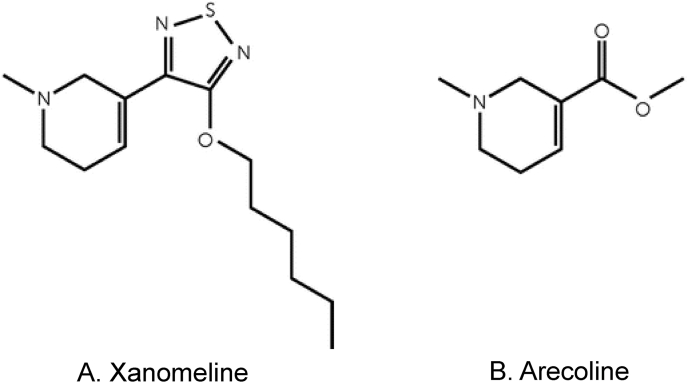
Chemical Structures of Xanomeline and Arecoline. Xanomeline (a) and arecoline (b) share a core scaffold that enables engagement of acetylcholine receptors. Xanomeline incorporates additional substituents, including an extended side chain and aromatic moieties, which confer greater subtype selectivity (M_1_/M_4_ receptor preference) and improved pharmacokinetic properties compared with the simpler arecoline structure.

With this background in mind, scientists at Eli Lilly developed the direct-acting (i.e., orthosteric) mAChR agonist xanomeline (Fig. 1a). Xanomeline is a small, lipophilic molecule belonging to the thiadiazole class of compounds. Its structure consists of a thiadiazole ring core that serves as a rigid scaffold, linked to a pyridine-derived moiety that mimics features of ACh binding, and a hexoxy side chain that extends outward to interact with hydrophobic regions of the receptor [34]. This structural arrangement enables xanomeline to orient within the mAChR's orthosteric binding pocket while also engaging nearby sub-pockets that contribute to its functional subtype preference [35].

Xanomeline is a synthetic derivative of the natural mAChR agonist arecoline (Fig. 1b), the most abundant psychoactive alkaloid in the betel nut (*Areca catechu*) [36]. Arecoline is a potent but relatively nonselective ACh receptor agonist that acts at both nAChR and mAChR families and has long been recognized for its central cholinergic effects (see Ref. [37] review). The betel nut is traditionally chewed by indigenous populations in Southeast Asia as a cultural practice [38,39], and interestingly, epidemiological studies have reported a lower incidence of psychosis and schizophrenia among habitual betel nut users [40,41]. Building on this pharmacological starting point, medicinal chemistry efforts at Eli Lilly and Novo Nordisk modified the arecoline scaffold to improve lipophilicity, brain penetration, and subtype preference, ultimately leading to the discovery of xanomeline [42,43]. Compared with its natural precursor, xanomeline retains robust mAChR agonist activity but displays greater functional selectivity for M_1_ and M_4_ receptors [44] and achieves a uniquely high brain-to-plasma ratio [45], making it well-suited for potentially treating central nervous system (CNS) disorders.

As mentioned, Eli Lilly's primary interest was to study xanomeline in AD patients to determine whether it would improve cognition and be safe and well tolerated [46]. To this end, Eli Lilly conducted a large, four-arm Phase 2 RCT in 343 patients with mild to moderate AD, in which patients received daily treatment with xanomeline at 75 mg, 150 mg, or 225 mg, or placebo, for six months. The study revealed several important findings. First, xanomeline produced a dose-dependent but modest improvement in cognition (measured by the Alzheimer's Disease Assessment Scale–Cognitive Subscale), with the largest effect observed at the 225 mg dose. However, this cognitive benefit emerged only after 8–10 weeks of treatment and was only observed in patients who completed the trial, as dropout rates were high due to undesirable adverse effects (AEs). Second, in this elderly population, xanomeline was not well tolerated, producing dose-dependent AEs, mostly gastrointestinal (GI; nausea and vomiting) in nature. Although most events were mild to moderate in severity, they were often persistent and led to discontinuation of treatment; approximately 50% of patients in the high-dose arm dropped out of the study due to these AEs. Finally, and most importantly, a completely unanticipated observation emerged; in AD patients who also had disruptive behavioral symptoms, including psychotic symptoms (delusions and hallucinations), now referred to as dementia-related psychosis, the investigators observed a marked and early improvement following just 2–3 weeks of xanomeline treatment. Xanomeline treatment also significantly and dose-dependently reduced the emergence of psychotic symptoms as well as agitation compared with placebo over the course of the six-month trial. Although these findings were post hoc in nature, they were all highly statistically significant and clinically meaningful [46].

The serendipitous clinical findings of improvement in psychosis and agitation symptoms in patients with AD treated with xanomeline opened a new avenue of research into the role of mAChRs in neuropsychiatric symptom domains. During this period, mAChRs, particularly the M_1_ receptor, were traditionally regarded as “cognition” receptors. The unexpected observation that a mAChR agonist could ameliorate psychotic and behavioral symptoms challenged a long-standing assumption and expanded the functional landscape of mAChR biology. The effects of xanomeline in “treating” and potentially “preventing” psychotic and other disturbing behavioral symptoms in patients with AD prompted extensive preclinical investigation at Eli Lilly, other pharmaceutical companies, and academia, aimed at uncovering the role of mAChRs implicated in these symptom domains. This work will be summarized briefly below but in essence in these preclinical models xanomeline shares many of the behavioral and electrophysiological properties of APDs, but it does not directly block DA D_2_ receptors [42], act in midbrain motor regions, or cause catalepsy [47], an animal model predictive of human EPS [48]. Collectively, these preclinical and clinical data prompted a small proof-of-concept study in patients with schizophrenia carried out at Indiana University [49]. In this 4-week study, 20 acutely psychotic patients with chronic schizophrenia were administered xanomeline (titrated over one week to 225 mg daily) or placebo. Despite the small size and relatively short duration, patients treated with xanomeline showed a marked improvement in psychotic symptoms, as measured by the Brief Psychiatric Rating Scale and PANSS total score, with clinical improvement evident as early as two weeks. Moreover, improvement in negative symptoms as well as cognition were also observed. While patients in this trial experienced cholinergic GI AEs (e.g., nausea and vomiting), they did not exhibit sedation, weight gain or EPS/akathisia [49], similar to what was observed in the trial of elderly AD patients [46].

Taken together, the two Eli Lilly-sponsored RCTs, the first initially demonstrating the efficacy of xanomeline in treating dementia-related psychosis in AD [46] and the second showing improvements in psychotic symptoms, negative symptoms and cognition in patients with schizophrenia [49], provided strong evidence that an mAChR agonist devoid of DA D_2_ receptor blocking activity could effectively treat psychosis and may also potentially improve the negative and cognitive symptoms of schizophrenia. The ongoing development and clinical evaluation of xanomeline will be described in subsequent sections of this manuscript.

## mAChR Biology: Structure and Function

mAChRs are class A (rhodopsin-like) G-protein–coupled receptors that share the conserved seven-transmembrane (TM) α-helical structure typical of this superfamily [50]. These helices are connected by extracellular and intracellular loops, which contribute to ligand recognition and G-protein coupling. The receptors contain two principal classes of binding sites: the orthosteric site, located deep within the TM domain and highly conserved across all five subtypes (M_1_–M_5_), and allosteric sites, located within the extracellular vestibule and at receptor–protein interfaces [50]. Orthosteric ligands, including ACh, directly activate the receptor, whereas allosteric modulators fine-tune receptor activity by altering the binding or signaling properties of orthosteric ligands [51]. The structural framework of the seven TM helices not only defines the orthosteric and allosteric binding sites but also shapes G-protein coupling specificity.

The five mAChR subtypes differ in tissue distribution, signaling partners, and functional roles. Broadly, M_1_, M_3_, and M_5_ receptors couple to G_q/11_ proteins, leading to activation of phospholipase C and downstream mobilization of intracellular calcium (Ca^2+^). By contrast, M_2_ and M_4_ receptors couple primarily to G_i/o_ proteins, which inhibit adenylyl cyclase, reduce cyclic adenosine monophosphate (cAMP) production, and modulate ion channel activity through G_βγ_ subunits [52]. However, it is now clear that mAChRs also engage alternative signaling cascades, including β-arrestin pathways and modulation of ion channels [53]. Biased signaling (functional selectivity) refers to the ability of a ligand to preferentially activate one signaling pathway over another at the same receptor [54].

This concept has been demonstrated across mAChR subtypes. At the M_1_ receptor, certain agonists show bias for Ca^2+^ mobilization via G_q_/phospholipase C over β-arrestin recruitment [55]. Xanomeline, while initially developed as a non-selective orthosteric agonist, demonstrates a functional preference for M_1_ and M_4_ receptor mediated signaling relative to M_2_ and M_3_ receptor pathways [44] and preferentially signals away from Ca^2+^ mobilization compared to Gα_i2_ [56]. At the M_4_ receptor, ligands such as VU0152100 [57,58] and related allosteric modulators [59] developed at Vanderbilt University show preferential bias toward G_i/o_-mediated inhibition of striatal DA release, sparing other pathways less relevant to antipsychotic-like efficacy. From a therapeutic standpoint, biased mAChR signaling allows for fine-tuning receptor responses; enhancing beneficial pathways (e.g., cortical excitation via M_1_ receptors, striatal regulation via M_4_ receptors) while avoiding those linked to AEs (e.g., β-arrestin-driven desensitization) [53]. As such, modern drug discovery is increasingly focused not only on subtype selectivity but also on engineering signaling bias to optimize efficacy and tolerability [[60], [61], [62]].

## Neural-Based Mechanisms of Schizophrenia: Roles of M_1_ and M_4_ Receptors

### Positive symptoms

The positive symptoms of schizophrenia are thought to arise from two primary mechanisms: increased dopaminergic transmission within the striatum and a hyperglutamatergic state in cortical–striatal circuits [63]. These represent the main hypotheses of schizophrenia pathophysiology; however, several additional neurotransmitter systems, including gamma-aminobutyric acid (GABA), serotonin, and neuropeptides, are also dysregulated and likely contribute to disease pathophysiology. Within this neurochemical framework, M_1_ and M_4_ receptors serve as critical modulators that restore balance across dopaminergic and glutamatergic pathways.

**Fig. 2:**
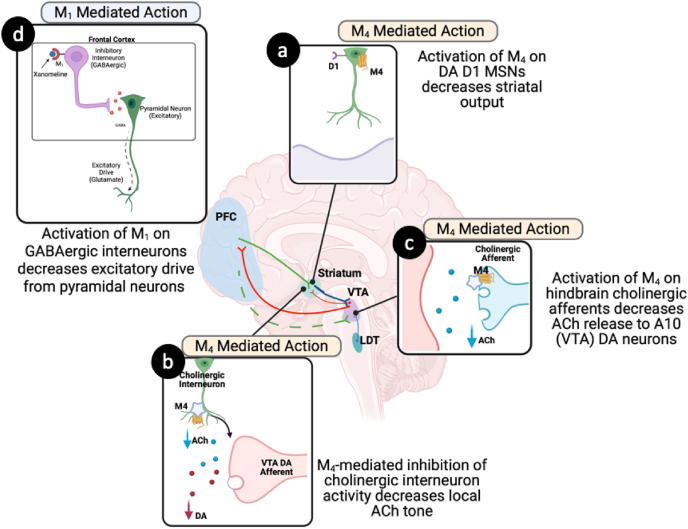
M_1_ and M_4_ Receptor–Mediated Regulation of Neural Circuits Underlying Psychotic Symptoms. Activation of M_1_ and M_4_ receptors decreases dopamine (DA) release through coordinated actions across cortical and subcortical circuits. M_4_ autoreceptors on cholinergic projections from the laterodorsal tegmental (LDT) nucleus that synapse onto DA neurons within the ventral tegmental area (VTA; c). Within the striatum, M_4_ receptors are localized on cholinergic interneurons, where they reduce terminal DA release by dampening interneuron activity (b). M_4_ receptors are also expressed on DA-D_1_ medium spiny neurons, where they decrease neuronal excitability and downstream output (a). In contrast to the broad effects of M_4_ receptor activation, M_1_ receptors are primarly expressed in the frontal cortex, where they reduce pyramidal neuron (glutamate) activity indirectly by enhancing inhibitory interneuron signaling (d). Abbreviations: ACh = acetycholine; MSNs = medium spiny neurons; VTA = ventral tegmental area.

M_4_ receptors play a key role in regulating dopaminergic activity through potentially multiple striatal and brainstem circuits (Fig. 2a–c). In the striatum, M_4_ receptors are enriched on medium spiny neurons (MSNs) of the direct pathway, where they co-localize with DA D_1_ receptors (Fig. 2a) [64]. Normally, DA D_1_ receptor activation enhances excitatory drive, promoting further DA release. M_4_ receptor activation counterbalances this effect via G_i/o_-coupled inhibition of adenylyl cyclase, dampening DA D_1_ receptor mediated excitability, and reducing feedback onto dopaminergic terminals [59,65]. This “cholinergic brake” limits hyperdopaminergic states. Presynaptic M_4_ autoreceptors on cholinergic interneurons also reduce ACh release, indirectly modulating DA neuron excitability in the striatum (Fig. 2b) [66]. Beyond the striatum, M_4_ receptors are expressed on cholinergic neurons of the laterodorsal tegmentum (LDT), which project directly to midbrain dopaminergic neurons in the ventral tegmental area (VTA) (Fig. 2c). Activation of M_4_ autoreceptors on these LDT neurons suppresses ACh release into the VTA, thereby reducing excitatory drive onto dopaminergic neurons [67,68]. This additional pathway provides a powerful mechanism for M_4_ receptor activators to stabilize mesolimbic DA output at its source, complementing the local striatal control of DA release.

M_1_ receptors play a distinct but complementary role in cortical regulation of DA signaling (Fig. 2d). M_1_ receptors are highly expressed in the frontal cortex, particularly in layer II/III inhibitory interneurons [69], where they act as gatekeepers of glutamatergic transmission projecting to midbrain DA systems [68]. M_1_ receptors couple to stimulatory G-proteins (G_q/11_). When activated, they enhance interneuron (GABAergic) firing, which in turn increases inhibitory tone onto cortical pyramidal neurons [69,70]. This strengthened inhibitory control reduces excessive glutamate release from pyramidal cells, thereby modulating downstream excitatory drive into the VTA and striatum [67,68].

Preclinical models provide converging behavioral evidence for these mechanistic roles. Psychostimulants (i.e., amphetamine) or N-methyl-d-aspartate (NMDA) receptor antagonists reliably induce locomotor hyperactivity, mimicking mesolimbic DA overdrive observed in patients. mAChR activation attenuates these effects (see Ref. [67] for review). The M_1_ and M_4_ preferrjng agonist xanomeline attenuates hyperlocomotion induced by both amphetamine and NMDA receptor antagonists [47,71], consistent with its capacity to dampen DA release via actions in cortical and subcortical circuits. Similarly, M_4_ receptor allosteric modulators suppress hyperlocomotor activity induced by psychostimulants [[57], [58], [59],^72^,^73^], demonstrating that M_4_ receptor engagement alone can normalize hyperdopaminergic states. In addition, M_4_ receptor activation can normalize behavioral, electrophysiological and neurochemical abnormalities associated with NMDA receptor hypofunction [57,74].

M_1_ receptors also contribute to the regulation of locomotor activity, though their effects appear dependent on ligand bias. Biased M_1_ receptor agonists such as TBPB (which preferentially engages β-arrestin–independent G_q/11_ signaling) and M_1_ receptor allosteric modulators like PQCA have been reported to attenuate psychostimulant-induced hyperlocomotion [75,76]. These findings suggest that M_1_ receptor activation within cortical circuits can indirectly constrain locomotor hyperactivity, likely by rebalancing cortical glutamatergic output onto striatal and midbrain DA pathways. Importantly, the ability of certain M_1_ receptor ligands to modulate locomotor readouts highlights that not all M_1_ receptor targeting compounds produce equivalent effects, and functional selectivity may be critical for optimizing therapeutic efficacy while minimizing side effects [77].

### Cognitive symptoms

The frontal cortex and hippocampus are two brain regions consistently implicated in the cognitive impairments observed in schizophrenia. The frontal cortex is critical for higher-order functions such as working memory, attention, and decision-making, while the hippocampus plays a central role in learning and memory by regulating the encoding and retrieval of information [33]. Dysregulation within these circuits is thought to contribute to the difficulties patients experience with organizing thoughts, retaining new information, and adapting to changing demands. Importantly, while these regions are strongly associated with cognitive dysfunction in schizophrenia, additional disrupted neural networks that include other cortical and subcortical structures are likely involved [78].

**Fig. 3:**
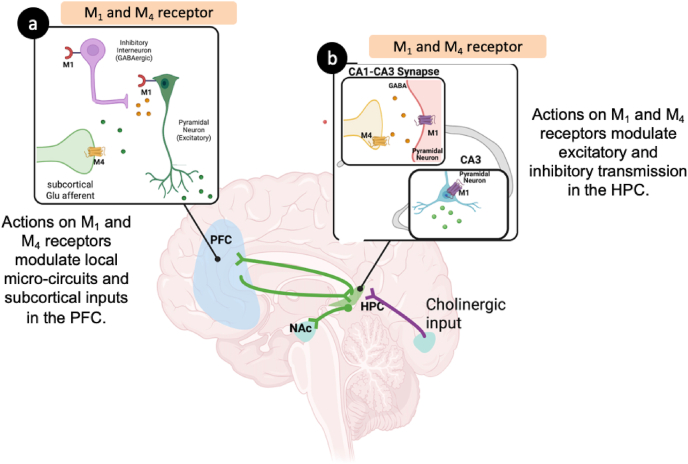
M_1_ and M_4_ Receptor Activation Restores Excitatory–Inhibitory Balance. M_1_ and M_4_ receptors play complementary roles in regulating cortical-hippocampal circuits that support cognitive function. In the frontal cortex (a) and hippocampus (b), M_1_ receptors are expressed on both inhibitory interneurons and layer V pyrmidal neurons, where they enhance inhibitory tone while constraining pyramidal (glutamate) cell excitability, thereby restoring excitatory-inhinitory balance. In parallel, M_4_ receptors are localized on glutamaterfic projection neurons in these regions (a,b), where their activation decreases excitatory drive and dampens downstream output. Together, these mechanisms normalize circuit dynamics critical for cognitive processing. Abbreviations; HPC = hippocampus; PFC = prefrontal cortex; NAc = nucleus accumbens.

M_1_ receptors exert powerful control over cortical and hippocampal circuits by regulating neuronal excitability, synaptic plasticity, and information filtering [33]. In the frontal cortex, M_1_ receptor activation can drive both excitatory and inhibitory responses within the same neuron, depending on intracellular signaling states, which allows for flexible modulation of working memory and attentional processes. M_1_ receptor activation is also layer-specific. Pyramidal neurons in superficial and deep layers show distinct cholinergic responsiveness, likely due to Ca^2+^ activated potassium channels that shape firing output (see Ref. [33] for review) (Fig. 3a). Beyond this, M_1_ receptors facilitate ACh release locally and interact with DA signaling, particularly DA D_1_ receptor pathways in pyramidal cell dendrites [79], to optimize prefrontal computations needed for cognitive flexibility. In the hippocampus, M_1_ receptors play a parallel role by modulating GABAergic interneurons that filter pyramidal cell firing and by activating second messenger pathways required for long-term potentiation [80] (Fig. 3b). Through these mechanisms, M_1_ receptor activation enhances the signal-to-noise ratio in both regions, ensuring that only relevant inputs are strengthened and encoded during learning and memory.

M_4_ receptors complement these actions by stabilizing excitatory drive and refining network output. In the frontal cortex, M_4_ receptors reduce excessive glutamatergic transmission at corticostriatal synapses [81], promoting long-term depression that gates downstream activity and regulates contextual representations (Fig. 3a). A similar mechanism operates in the hippocampus, where M_4_ receptor activation suppresses excitatory input between pyramidal neurons [74], preserving oscillatory rhythms that support accurate memory encoding (Fig. 3b). Across both regions, M_4_ receptors act as a brake on excitatory overdrive, preventing destabilization of network activity while coordinating circuit-level synchrony.

Cognitive impairments in schizophrenia, including deficits in working memory, attention, and executive function, are among the most disabling aspects of the illness and are predictive of poor long-term functional outcomes [8,9]. Preclinical models typically assess these domains using paradigms such as novel object recognition, radial arm maze, Morris water maze, attentional set-shifting, and operant-based measures of attention and working memory [82]. mAChR activation consistently improves performance across these cognitive tasks (see Ref. [33] for review). Dual M_1_/M_4_ receptor activators enhance working memory and attentional control in NMDA antagonist treated rodents [71,83,84]. M_1_ receptor agonists and allosteric modulators enhance recognition memory, attentional set-shifting, and learning flexibility [[85], [86], [87]], consistent with the role of the M_1_ receptor in driving prefrontal cortical excitability and hippocampal-dependent plasticity. M_4_ receptor modulation has also been implicated in cognition, with M_4_ receptor allosteric modulators improving performance in tasks requiring cognitive flexibility [88], likely through stabilization of striatal DA signaling that reduces “circuit noise.” These findings provide strong support for targeting M_1_ and M_4_ receptors as a strategy to address these cognitive symptoms, which remain poorly treated by current DA D_2_ receptor based APDs.

### Negative symptoms

Negative symptoms in schizophrenia, such as blunted affect, social withdrawal, and reduced motivation, have been linked to dysfunction in subcortical structures including the striatum, amygdala, and related limbic circuits [10]. The striatum is particularly relevant to reward processing and motivation, while the amygdala contributes to emotional reactivity and social behaviors. Alterations within these and other subcortical regions likely contribute to the diminished drive and affective flattening characteristic of negative symptoms. However, these structures are likely not the only contributors, and the underlying neural circuitry of negative symptoms is complex, reflecting a broad umbrella of clinical features that makes pinpointing specific neural mechanisms and regions especially challenging.

Within the striatum, M_1_ receptors regulate MSN excitability and synaptic plasticity [89,90], shaping how cortical and thalamic inputs are translated into motivational drive and goal-directed behavior. Disruptions in this regulation may impair DA and glutamate signaling, contributing to the reduced reinforcement learning and diminished initiation of action characteristic of negative symptoms. Similarly, in the amygdala and related limbic structures, M_1_ receptors influence excitatory–inhibitory balance and modulate emotional reactivity, social processing, and affective responsiveness [91,92]. Dysfunction of M_1_ receptor signaling within these interconnected networks may therefore disrupt both motivational and emotional domains, amplifying the broad and heterogeneous clinical features associated with negative symptoms (Table 1).

**Table 1:** M_1_ and M_4_ receptors regulate cortical, striatal, and amygdala circuits to mitigate negative symptoms. Activation of M_1_ receptors restores excitatory–inhibitory balance and modulates both DA D_1_ and DA D_2_ MSN excitability, while M_4_ receptor activation dampens DA D_1_ MSNs activity, induces LTD at corticostriatal synapses, and reduces glutamatergic output from the amygdala. Together, these mechanisms stabilize motivation, social engagement, and affective regulation.

Region	Receptor	Localization	Mechanism of Action	Impact on Circuitry	Relevance to Negative Symptoms
Striatum	M_1_	DA D_1_ and DA D_2_ MSNs	Modulates excitability of both MSN subtypes	Balances direct and indirect pathway output	Stabilizes striatal activity to improve motivation and goal-directed behavior
	M_4_	DA D_1_ MSNs	Reduces excitability of DA D_1_ MSNs	Dampens excessive direct pathway signaling	Normalizes reward and reinforcement processes
Frontal cortex	M_1_	Interneurons + pyramidal neurons	Enhances inhibition and reduces pyramidal overactivity	Restores excitatory–inhibitory balance	Improves executive function and cognitive flexibility
	M_4_	Corticostriatal glutamatergic projection neurons	Induces LTD at corticostriatal synapses	Reduces excitatory drive into striatum	Modulates corticostriatal signaling linked to apathy and social withdrawal
Amygdala	M_1_	Glutamatergic projection neurons	Restores excitatory neurotransmission	Normalizes affective input into cortical–striatal circuits	Supports emotional engagement and social interaction
	M_4_	Glutamatergic neurons in BLA	Reduces excitatory Glu output onto subcortical targets (striatum, VTA, PFC inputs)	Constrains hyperactive limbic signaling	Regulates emotional salience and stress reactivity, reducing withdrawal and affective blunting

BLA, basal lateral amygdala; DA, dopamine; Glu, glutamate; LTD, laterodorsal tegmental nucleus; MSN: medium spiny neurons; PFC, prefrontal cortex; VTA, ventral tegmental area.

M_4_ receptor activation has emerged as an important modulator of glutamatergic signaling within corticostriatal and limbic circuits [59,81], pathways strongly implicated in the development of negative symptoms in schizophrenia. By inhibiting excessive glutamate release at corticostriatal synapses, M_4_ receptors promote long-term depression and help filter cortical input before it drives downstream striatal and limbic activity (Table 1). This regulation prevents glutamatergic overdrive that can destabilize reward and motivational networks, processes central to the expression of anhedonia, amotivation, and social withdrawal. In this way, M_4_ receptor activation acts as a brake on excitatory transmission, restoring balance in corticostriatal pathways and potentially alleviating core features of negative symptoms.

Genetic studies also highlight the potential importance of M_4_ receptors in these behavioral domains. M_4_ receptor knockout mice display deficits in social interaction and impaired reward processing [93], suggesting that M_4_ receptor signaling is critical for motivational drive and social behavior. These M_4_ receptor knockout phenotypes are insensitive to M_4_ receptor PAMs, confirming receptor specificity. M_1_ receptors also contribute to negative symptom–like behaviors. For instance, M_1_ receptor knockout mice show impaired social recognition and altered affective responses [87], implicating cortical M_1_ receptor signaling in circuits governing social cognition and motivation. Pharmacologically, M_1_ receptor selective agonists enhance social recognition and mitigate anhedonia in reward-based tasks [94]. These preclinical findings support the potential clinical benefit of M_1_ and M_4_ receptor agonists in treating the negative symptoms of schizophrenia.

## From Preclinical Proof of Concept to Clinical Evaluation

While several mAChR modulators have advanced into clinical testing (discussed below), publicly available preclinical data for many of these compounds remain limited, with few disclosures describing in vitro pharmacology or in vivo efficacy profiles. Nonetheless, the translational rationale for this mechanism is grounded in a well-characterized body of preclinical research with tool compounds and earlier chemical series. Comparing in vitro potency, selectivity, and signaling profiles with in vivo efficacy and tolerability outcomes across mAChR agonists and modulators will help clarify the mechanistic role of individual receptor subtypes in schizophrenia and guide the identification of the pharmacologic features most predictive of clinical benefit.

Emraclidine is a highly potent and selective PAM of the M_4_ receptor, designed to achieve central efficacy without peripheral cholinergic activation. In cell-based assays, emraclidine produced a half-maximal effective concentration (EC_50_) of 12 nM at human M_4_ receptors and demonstrated >390-fold selectivity over M_2_, with negligible activity at M_1_, M_3_, and M_5_ receptors [95]. In contrast, reported EC_50_ values of xanomeline at human M_4_ receptors range from 200 to 300 nM [96,97]. In preclinical behavioral models relevant to psychosis, emraclidine produced a dose-dependent effect, with a minimum effective dose of 3.2 mg/kg (subcutaneous [sc]) and correlated with plasma exposures consistent with central M_4_ receptor engagement [95]. Xanomeline demonstrated robust antipsychotic-like efficacy in preclinical models, producing significant behavioral effects at doses as low as 1 mg/kg (sc) [98], supporting its ability to engage central mAChR targets even at relatively modest systemic exposures. The difference in EC_50_ values between xanomeline and emraclidine has important pharmacologic implications. A lower EC_50_ at M_4_ receptors reflects greater potency and more efficient receptor engagement, enabling effective central activation at lower doses and reducing the likelihood of peripheral receptor stimulation that contributes to AEs.

Another agent currently in clinical development, ML-007, has also been described in a recent preclinical publication characterizing its pharmacologic profile [99]. ML-007 exhibited EC_50_ values of 120 nM (human M_1_ receptor) and 830 nM (human M_4_ receptor), indicating moderate potency and a slight bias toward M_1_ receptor activity. Despite being less potent in vitro than the reference agonist xanomeline (human M_1_ receptor = 2.3 nM; human M_4_ receptor = 5.5 nM), ML-007 demonstrated ten-fold greater efficacy in preclinical models of psychosis (minimum efficacious dose ≈ 0.3 mg/kg, intraperitoneal [ip]), compared to 3–10 mg/kg xanomeline required for comparable effects. Pharmacokinetic analysis showed that ML-007 achieved a maximum concentration of drug (C_max_) of ∼115 ng/mL (≈700 nM) after 0.3 mg/kg ip dosing, whereas xanomeline's brain exposure was below quantifiable limits (<1.8 nM) following 3 mg/kg ip administration. The authors emphasized that these differences reflect higher brain bioavailability and free-drug exposure for ML-007 rather than intrinsic receptor potency. Importantly, xanomeline was administered ip rather than sc, a route known to yield lower systemic bioavailability compared to sc dosing used in prior studies; thus, the observed potency gap between the two compounds may partially reflect non-optimal pharmacokinetic sampling and administration route rather than fundamental efficacy differences.

In summary, direct comparison of mAChR agents based on available preclinical data is inherently challenging, as differences in assay formats, receptor systems, functional readouts, and dosing paradigms introduce substantial variability across studies. Divergent use of orthosteric versus allosteric assays, recombinant versus native receptor preparations, and differing in vivo behavioral models further complicate interpretation of potency, selectivity, and efficacy profiles. As a result, apparent distinctions between compounds may reflect methodological differences rather than true pharmacologic divergence. Ultimately, well-controlled clinical studies will be required to determine how these agents differ in terms of efficacy, tolerability, and functional outcomes. Until such data emerge, comparisons between mAChR therapeutics should be made cautiously, with recognition of the limits of cross-study preclinical benchmarking.

## Clinical Journey of mAChR Agents

### Development of xanomeline-trospium (COBENFY™) at Karuna Therapeutics

Despite encouraging data from the two Eli Lilly RCTs [46,49], the AE profile of xanomeline, driven by peripheral mAChR activation, precluded its further development. Around 2010, scientists at PureTech Health and Karuna Therapeutics devised a novel strategy to mitigate this AE profile in order to improve the drug's safety and tolerability. Given xanomeline's robust brain penetration [45], could its peripherally mediated AEs (e.g., nausea, vomiting, salivation, sweating) be reduced by combining it with a peripherally-restricted antimuscarinic agent? After an extensive search, trospium was selected. Trospium has been safely used for years to treat overactive bladder, primarily in women, and, as a quaternary salt, it does not readily cross the blood–brain barrier due to its polar structure [100]. This combination of xanomeline plus trospium was initially named KarXT, representing a mechanistically rational combination designed to balance efficacy and tolerability by leveraging central activation with peripheral blockade. In subsequent Phase 1 studies, doses and ratios of xanomeline and trospium were varied to optimize tolerability, and indeed, cholinergic AEs induced by xanomeline were reduced by approximately 50% when co-administered with trospium at the optimal ratios [101].

With these data in hand, Karuna Therapeutics advanced the development of xanomeline-trospium by conducting three registrational RCTs [[18], [19], [20]]. In each trial, dosing was titrated flexibly: all patients began with a low, therapeutically inactive dose (50/20 mg twice daily [BID]) for two days, which was then increased on day 3 to 100/20 mg BID. After one week at this intermediate dose, patients could be further increased to the highest dose (125/30 mg BID), depending on individual tolerability. Patients who experienced mild AEs were allowed to remain at the intermediate dose, while others could escalate to 125/30 mg BID. In this way, patients who were relatively slow metabolizers could still be maintained on a potentially effective dose (100/20 mg BID) with reduced risk of AEs. The one-week titration schedule in the EMERGENT RCTs was intended to help patients accommodate to pro-cholinergic AEs, a strategy also used with cholinesterase inhibitors [102].

EMERGENT-1 (NCT03697252) was a 5-week, inpatient, Phase 2 trial that randomized 180 acutely psychotic patients with schizophrenia (90 per arm) to xanomeline-trospium or placebo under blinded conditions. The primary endpoint was the change in PANSS total score from baseline to week 5, with secondary endpoints including the PANSS negative symptom subscale and the Clinical Global Impression–Severity (CGI-S) score. An exploratory cognitive battery was also administered to assess changes in cognitive function [20]. EMERGENT-2 (NCT04659161) [18] and EMERGENT-3 (NCT04738123) [19] were Phase 3 studies designed similarly to the initial Phase 2 trial but with larger sample sizes (approximately 120 patients per arm). Patients from both Phase 3 studies were eligible to roll over into an open-label extension to assess the long-term safety and efficacy of xanomeline-trospium [103]. Importantly, several key design features were incorporated into the EMERGENT program to help minimize placebo response. These included maintaining relatively large placebo groups in two-arm, flexible-dose trials, the use of remote raters to validate PANSS ratings, and limiting the number of participating trial sites, among other measures.

The results of the three EMERGENT RCTs were unambiguous. In all three studies, the primary endpoint was achieved, with a highly significant reduction in PANSS total score from baseline to endpoint (p < 0.0001). The observed effect sizes (Cohen's *d* = 0.61–0.71) establish xanomeline-trospium as a robustly effective APD in acutely psychotic patients with schizophrenia. Secondary endpoints were also met, with consistent improvement on the CGI-S across all trials and significant benefit on the PANSS negative symptom subscale in two of the three studies (i.e., EMERGENT 1 [20] and EMERGENT 2 [18]). The AE profile in EMERGENT-1 was largely consistent with mAChR activation but was substantially improved relative to historical xanomeline monotherapy. The most commonly reported AEs included nausea, constipation, vomiting, dyspepsia, and dry mouth, generally mild to moderate in severity and transient in duration. Discontinuation due to AEs occurred in approximately 10–12 % of patients in the xanomeline–trospium arm versus 6 % in placebo, and no significant EPS, metabolic, or prolactin-related adverse effects were observed. Across both Phase 3 studies, the safety and tolerability profile of xanomeline–trospium was favorable and consistent with prior findings. The most frequent AEs included nausea, constipation, vomiting, dyspepsia, dry mouth, and abdominal discomfort, with treatment-emergent AEs generally occurring early in therapy and diminishing over time. Rates of discontinuation due to AEs were modest (approximately 10–15%), and serious AEs were rare and comparable to placebo. Importantly, no evidence of weight gain, sedation, metabolic disturbance, or EPS, common liabilities of DA D_2_ receptor antagonists, was observed [[18], [19], [20]].

In addition to the pre-specified primary and secondary endpoints, several post-hoc exploratory outcomes were assessed in the EMERGENT RCTs. For example, in EMERGENT-2 and EMERGENT-3, cognition was evaluated using the Cambridge Neuropsychological Test Automated Battery (CANTAB) [104]. While overall improvement in cognition with xanomeline–trospium showed only a trend, approximately half of the enrolled patients performed within the “normal limits” of the test battery. Importantly, when patients were stratified by baseline cognitive performance, robust effects emerged: in those with poor baseline cognition (≥1.0 standard deviation [SD] below the mean of healthy controls), treatment with xanomeline–trospium produced a highly significant improvement compared with placebo (p < 0.001; Cohen's *d* = 0.52). In patients with more severe baseline impairment (≥1.5 SD below the mean), the effect size increased further (*d* = 0.81). Notably, improvements in cognition were only weakly correlated with changes in PANSS total score, suggesting that the cognitive benefit was not simply a byproduct of overall symptomatic improvement [104]. Similar post-hoc findings were observed for negative symptoms, where the degree of improvement depended on baseline severity [105]. Although exploratory, these results raise the possibility that xanomeline–trospium may address all three core symptom domains of schizophrenia (positive, negative, and cognitive). However, further studies in patients with stable positive symptoms and predominant cognitive or negative symptoms are needed to confirm this potential. It should also be emphasized that the EMERGENT trials were relatively short (5 weeks in duration), and the full magnitude of benefit may not have been captured. Nonetheless, taken together, these clinical data, spanning primary, secondary, and exploratory endpoints, strongly support the novel therapeutic profile of this new class of mAChR medicines for schizophrenia and, potentially, dementia-related psychosis.

### COBENFY™ Data and development at Bristol Myers Squibb

#### Adjunctive Phase 3 trial (ARISE)

In a Phase 3 (NCT06309200) 6-week, double-blind, randomized, placebo-controlled, multicenter outpatient study, adults aged 18–65 years with schizophrenia who remained symptomatic despite treatment with a stable background atypical antipsychotic (PANSS ≥70 at screening) were randomized to adjunctive COBENFY or placebo. Although the study did not meet its primary endpoint (least-squares [LS] mean change in PANSS total score at Week 6: −14.3 vs −12.2 for placebo; LS mean difference −2.0, 95 % CI –4.5 to 0.5; p = 0.11), post hoc subgroup analyses revealed a nominally significant benefit among patients receiving non-risperidone background therapy (n ˜ 130), with a mean PANSS reduction of −15.1 for COBENFY versus −11.7 for placebo (LS mean difference −3.4; 95 % CI –6.3 to −0.5; p ≈ 0.03) [106]. In contrast, no meaningful difference was observed among patients maintained on risperidone (n ˜ 60), a finding that may reflect a floor effect; that is, limited capacity for further improvement in patients already demonstrating strong dopaminergic blockade and lower baseline symptom severity at randomization.

Safety and tolerability in ARISE were consistent with the established COBENFY profile, with the most frequent treatment-emergent adverse events being nausea, dyspepsia, constipation, vomiting, abdominal discomfort, diarrhea, dizziness, tachycardia, and mild increases in blood pressure. Most events were mild to moderate and transient, and no new safety signals emerged relative to prior studies [106]. Taken together, the ARISE findings suggest that while COBENFY did not achieve a statistically significant benefit in the overall adjunctive population, the signal observed in patients receiving non-risperidone background therapy warrants further investigation to determine whether mAChR activation may differentially augment antipsychotic response depending on the underlying pharmacologic mechanism of the concomitant DA D_2_ receptor targeted agent.

## Other mAChR Agents in Development

Several other orthosteric and allosteric mAChR agonists are advancing through clinical development, reflecting the resurgence of interest in cholinergic pharmacology for potentially treating schizophrenia and other neuropsychiatric disorders. These efforts include both M_1_ and M_4_ receptor-preferring orthosteric agonists as well as allosteric modulators designed to achieve greater subtype selectivity and improved tolerability compared with earlier dual M_1_/M_4_ receptor orthosteric agonists. Together, these programs highlight a diverse pipeline of mAChR-targeted therapeutics that build upon the clinical validation established by xanomeline-trospium, each aiming to refine efficacy, and reduce side-effect liability through advances in receptor selectivity and pharmacological precision.

### mAChR compounds in clinical development

Emraclidine, a M_4_ receptor PAM, was evaluated in a Phase 1b (NCT04136873) and two Phase 2 trials (EMPOWER-1 [NCT05227690] and EMPOWER-2 [NCT05443724]) as a once-daily (QD) oral monotherapy for acute schizophrenia. While the Phase 1b study showed encouraging results, both the efficacy and safety data were impressive [107], Phase 2 studies unfortunately did not meet their primary efficacy endpoint on the PANSS total score [108]. Importantly, emraclidine demonstrated a favorable safety and tolerability profile, with only mild to moderate AEs reported [107,109], supporting the overall safety and good tolerability achieved by targeting M_4_ receptors. While the Phase 2 results with emraclidine were disappointing from an efficacy standpoint, it is unclear exactly why these Phase 2 RCTs failed. Although there was a numerical separation on the PANSS total score between the emraclidine and placebo arms, the differences did not reach statistical significance. Abbvie has recently announced that they will continue to pursue the development of emraclidine by launching new Phase 1 multiple ascending dose [110] and Phase 2 dose-ranging studies (NCT07145918), exploring higher doses of emraclidine than previously studied. It is possible that inadequate doses of emraclidine were studied in their earlier Phase 2 studies and (or) clinical trial execution proved problematic. From both a clinical and a scientific perspective the results of this Phase 2 trial will be important.

Neurocrine Biosciences has advanced direclidine (NBI-1117568), a selective M_4_ receptor orthosteric agonist, into clinical development for schizophrenia. In a randomized, placebo-controlled Phase 2 study, the 20 mg QD dose of direclidine demonstrated a statistically significant reduction in PANSS total score compared to placebo (placebo-adjusted change of −7.5 points at week 6, p = 0.011; effect size ≈ 0.61) [111]. Secondary endpoints, including the Clinical Global Impression of Severity (CGI-S) and Marder factor scores for both positive and negative symptoms, also showed improvement. Notably, higher doses (40 mg QD, 60 mg QD, and 30 mg BID) while numerically better than placebo did not achieve statistical significance [111], raising questions about the optimal therapeutic window. Treatment with direclidine was generally well tolerated, with AE rates similar to placebo [111]. Reported side effects included mild somnolence, dizziness, and transient heart rate elevations, but there were no significant metabolic effects or weight gain [111], differentiating the compound from traditional APDs and aligning with the favorable safety profile expected for M_4_ receptor pharmacology. Encouraged by the Phase 2 results, Neurocrine has initiated a global Phase 3 program enrolling approximately 280 acutely psychotic patients with schizophrenia [111]. These studies will be critical to confirm the efficacy of direclidine, clarify dose dependency, and determine whether selective M_4_ receptor agonism alone can achieve clinically meaningful benefits across symptom domains.

ANAVEX3-71 is a novel investigational compound that combines sigma-1 receptor agonism with allosteric agonism of the M_1_ receptor [112]. A Phase 2 placebo-controlled study (NCT06245213) is currently underway in the US, with 71 participants enrolled across two parts: a multiple ascending dose safety arm (Part A) and a 28-day efficacy arm (Part B). Preliminary data from Part A demonstrated dose-dependent engagement of neurophysiological biomarkers, including enhanced 40 Hz auditory steady-state response coherence and increased resting-state alpha power, both linked to cortical function and symptom domains in schizophrenia. Importantly, the compound was well tolerated, with no serious AEs reported [113]. Topline efficacy results from Part B are anticipated in the second half of 2025. If confirmed, ANAVEX3-71 would represent the first dual sigma-1/M_1_ receptor agonist to demonstrate clinical benefit in schizophrenia, highlighting the potential of integrated sigma-1 and cholinergic activity and neuroprotective mechanisms as a next-generation therapeutic strategy.

MapLight Therapeutics is developing ML-007C-MA, an oral formulation that combines a dual M_1_/M_4_ mAChR agonist (ML-007) with a peripherally-restricted mAChR antagonist, designed to reduce unwanted peripheral cholinergic side effects while preserving central mAChR activity. This approach parallels the xanomeline–trospium strategy. In a Phase 1 program involving 82 healthy adult and elderly participants, ML-007 co-formulated with its peripheral antagonist was well tolerated, with safety and pharmacokinetic profiles supportive of QD or at most BID dosing [114]. Importantly, cerebrospinal fluid sampling confirmed central exposure consistent with anticipated therapeutic levels, while peripheral side effects were effectively suppressed by the antagonist [115]. Based on these results, MapLight recently initiated the ZEPHYR Phase 2 trial, a randomized, placebo-controlled study enrolling patients with acute exacerbations of schizophrenia, with primary efficacy measured by change in PANSS total score at week 5 [116].

Syremis Therapeutics is also developing a dual M_1_/M_4_ receptor agonist (ST-905). This compound is extremely potent and designed to be dosed QD. Based on preclinical studies, ST-905 closely phenocopies xanomeline-trospuim but with improved drug-like properties such as good oral bioavailability and high potency making it suitable for a long-acting injectable (LAI) [117].

NS-136, developed by NeuShen Therapeutics, is a selective M_4_ receptor allosteric modulator currently in a first-in-human Phase 1 trial in healthy volunteers. The ongoing Phase 1 study is evaluating safety, tolerability, and pharmacokinetics following single and multiple ascending oral doses, with early reports suggesting predictable pharmacokinetics and a favorable tolerability profile [118]. Pending successful outcomes, NS-136 is expected to advance into patient-based studies, where it may provide a differentiated treatment option targeting positive, negative, and cognitive symptoms while avoiding the liabilities of DA D_2_ receptor antagonists.

Terran Biosciences is a biotechnology company focused on developing novel therapeutics and technologies for CNS disorders. Building on the strong efficacy and safety signals demonstrated by xanomeline–trospium in Phase 3 trials for schizophrenia, Terran has designed proprietary oral QD and LAI TerXT formulations to improve pharmacokinetics, tolerability, and adherence [119]. Clinical data from Terran remain pending, however, and the therapeutic profile of TerXT will require confirmation in upcoming trials.

## Future Directions and Unanswered Questions

### Pharmacological principles of mAChR modulation

Recent advances in mAChR pharmacology have emphasized not only the importance of receptor subtype selectivity, but also how certain receptor subtypes are engaged. For the M_1_ receptor, distinctions between partial and full agonist activity appear highly relevant to balancing efficacy and tolerability [120]. Traditional orthosteric agonists such as xanomeline display near full agonist efficacy at the M_1_ receptor as well as biased downstream signaling via G_s_, produce robust central effects but also dose-limiting peripheral cholinergic side effects - this has driven the development of novel biased agonists designed to fine-tune M_1_ receptor activity and broaden the therapeutic window.

At the M_4_ receptor, a parallel shift in focus has also centered on biased signaling properties. While M_4_ receptors canonically couple to G_i/o_ proteins to reduce cAMP levels and inhibit DA D_1_ receptor–driven striatal output, M_4_ receptors can also engage alternative pathways, including ion channel modulation and β-arrestin–mediated signaling [53]. Preclinical studies suggest that ligands which bias toward G_i/o_-mediated inhibition of hyperdopaminergic signaling are most effective at reducing psychosis-related behaviors, whereas broader signaling engagement may increase the risk of side effects [53]. This has positioned signaling bias at the M_4_ receptor as a critical design principle, complementing the partial agonism strategy at the M_1_ receptor.

Together, these insights highlight a paradigm shift in mAChR drug discovery: away from simple “on/off” receptor activation, and toward precision pharmacology that leverages receptor selectivity, partial versus full agonism, signaling bias, and orthosteric versus allosteric modulation to optimize efficacy and improve tolerability. Such strategies will likely be central to the development of next-generation mAChR therapeutics for schizophrenia and other neuropsychiatric disorders.

The intricacies of mAChR activation and signaling outlined above make these receptors attractive targets for novel drug discovery. However, several critical questions remain unanswered, and the design of next-generation mAChR activator for schizophrenia will be challenging until the optimal pharmacological properties, balancing efficacy and tolerability, are better defined. For example: exactly which mAChR subtypes are required for optimal efficacy? Will a selective M_4_ receptor orthosteric or allosteric activator be sufficient to treat positive symptoms? Will allosteric modulators, which rely on orthosteric activation by endogenous ACh, be as effective as direct-acting orthosteric agonists? The AE profile of M_4_ receptor selective orthosteric and allosteric agonists also remains incompletely characterized, particularly at therapeutically relevant doses. The role of M_1_ receptor activation is similarly unresolved: while peripheral M_1_ receptors likely mediate many of the GI AEs observed with dual M_1_/M_4_ receptor agonists [121,122], M_1_ receptors may also contribute to the efficacy reported for xanomeline–trospium. If so, do M_1_ receptors influence improvement in positive, negative, or cognitive symptoms? Put differently, will the “dirtier” dual M_1_/M_4_ receptor agonists prove more effective across a broader range of symptoms than highly selective compounds, despite their associated side effects? Conversely, can more selective M_4_ receptor agonists deliver comparable efficacy with fewer GI liabilities? What role might partial agonists at the M_1_ receptor and/or M_4_ receptor play? Finally, xanomeline exhibits weaker but significant activity at other mAChRs, such as the M_3_ receptor [123], does this contribute meaningfully to its clinical effects? Answering these questions will require more definitive results from ongoing clinical trials, as well as insights from new compounds that emerge in development.

In addition to the receptor-mediated determinants of efficacy and tolerability, it's important to consider the optimal drug-like properties of the various mAChR compounds being advanced. Xanomeline-trospium, the first of this new class of APD, requires BID dosing and has a significant “food effect” which complicates its use in real-world settings. Ideally, a QD dosing regimen without a food effect would be highly advantageous. The label for xanomeline-trospium also has appropriate warnings for both hepatic and genitourinary AEs that were observed mostly in susceptible populations of patients [124]. The latter appear to be unique to xanomeline or trospium and/or to the mechanism of action of the two-drug combination acting at the various mAChRs. Finally, ideally the development of a LAI formulation for any effective and safe mAChR agonist in this patient population is highly desirable and if achievable will clearly compliment the oral formulations. However, the pharmacological requirements for LAIs (e.g., high potency, low water solubility, and long half-life) are often different than those for oral formulations and not all mAChR agonists currently being developed are amendable to a LAI.

The translation of mAChR pharmacology into effective treatments for schizophrenia depends not only on the properties of the compounds themselves but also on how they are evaluated in clinical settings. Lessons from recent and ongoing clinical trials highlight that patient selection, illness stage, background therapy, and trial design are critical determinants of success. Because mAChR agents act through M_1_ and M_4_ receptor modulation of cortical–striatal–midbrain circuits rather than DA D_2_ receptor antagonism, they may benefit distinct patient subgroups and thus may require novel trial frameworks to capture their therapeutic signal.

## Biomarkers of mAChR Engagement

Growing evidence suggests that a subset of individuals with schizophrenia exhibit a mAChR deficit, and several emerging in vivo biomarkers that may identify patients most likely to benefit from mAChR directed therapies rather than traditional DA D_2_ receptor antagonists. Non-response to dopaminergic antipsychotics, coupled with specific symptom clusters, such as marked cognitive impairment, visual hallucinations, and disorganization, may signal cholinergic dysfunction consistent with a “hypomuscarinic” subtype. Electrophysiologic measures such as reduced mismatch negativity (MMN), which is sensitive to mAChR but not DA D_2_ receptor modulation, provide a promising functional biomarker of mAChR signaling deficits (see Ref. [125]). This biomarker may support a model in which impaired mAChR function underlies symptoms in a biologically distinct subgroup, offering a rational framework for precision stratification in future clinical trials of mAChR activators.

A recent study by Halassa provides valuable real-world evidence supporting the therapeutic relevance of mAChR modulation in psychosis. In two inpatient cohorts (n = 49), adjunctive COBENFY produced meaningful improvements in a subset of patients, with negative symptoms and stimulant-associated psychosis emerging as the strongest predictors of response, which may reflect greater sensitivity of M_1_ and M_4_ receptor regulated cortico-striatal circuits. In contrast, patients with intellectual disability were consistently nonresponsive, suggesting cholinergic pathway disruption may limit benefit [126]. These findings highlight the possibility of biologically distinct psychosis subgroups shaped by differences in mAChR function and underscore how mAChR agonists can “unmask” latent negative symptoms through improvements in social engagement. For drug development, the study reinforces mAChR modulation as a mechanistically distinct avenue for antipsychotic therapy and emphasizes the importance of precision approaches, including biomarker-guided patient selection and trial enrichment strategies tailored to mAChR responsive phenotypes.

## Conclusion

mAChR based pharmacology represents a paradigm shift in the treatment of schizophrenia. Unlike conventional DA D_2_ receptor antagonists, which primarily reduce positive symptoms but often with troublesome side effects and leave negative and cognitive symptom domains largely untreated [127], mAChR orthosteric agonists and allosteric modulators act through distinct cortical–striatal–midbrain pathways. By engaging M_1_ and M_4_ receptors, these compounds may normalize glutamatergic and dopaminergic transmission, offering the potential for broader and more balanced symptom control.

Clinical trial data with xanomeline, particularly in combination with the peripherally-restricted mAChR antagonist trospium, provide compelling proof-of-concept for this approach, demonstrating robust antipsychotic efficacy with emerging signals of benefit on negative and cognitive symptoms [[18], [19], [20], [21],^105^,^128^]. These results validate mAChR modulation as a clinically relevant mechanism distinct from DA D_2_ receptor blockade. In parallel, the development of highly selective orthosteric and allosteric agonists for M_1_ and M_4_ receptors aim to refine therapeutic benefits across symptom domains and improve tolerability.

The excitement surrounding mAChR therapies extends beyond pharmacology as it reflects the possibility of transforming patient lives. By moving beyond the constraints of DA D_2_ receptor antagonism, mAChR-based compounds may reduce treatment burden and improve cognition and motivation in order to restore functional outcomes that matter most to patients and families. Future work integrating biomarkers, patient stratification, and precision pharmacology will be essential to fully realize this promise. Together, these advances highlight mAChR modulation as a promising approach to potentially reshape the therapeutic landscape of schizophrenia.

## Author contributions

SMP and SEY contributed to conceptualization, resources, original writing, and review and editing. SEY created the figures and tables.

## Declaration of competing interest

The authors declare the following financial interests/personal relationships which may be considered as potential competing interests: Steven Paul reports a former relationship with Karuna Therapeutics Inc that includes: board membership and equity or stocks. Steven Paul reports a relationship with SAGE Therapeutics Inc that includes: former board membership and equity or stocks. Steven Paul reports a relationship with Voyager Therapeutics Inc that includes: former board membership and equity or stocks. Steven Paul reports a relationship with Rapport Therapeutics Inc that includes: board membership and equity or stocks. Steven Paul reports a relationship with Seaport Therapeutics Inc that includes: board membership and equity or stocks. Steven Paul reports a relationship with Alnylam Pharmaceuticals Inc that includes: equity or stocks. Steven Paul reports a relationship with Eli Lilly and Company that includes: equity or stocks. Samantha Yohn reports a former relationship with Karuna Therapeutics Inc. Samantha Yohn reports a relationship with Neurocrine Biosciences that includes: equity or stocks.
